# Metabolite-GEN: integrated prediction of sites of metabolism, reaction class, and metabolite structures using graph edit networks

**DOI:** 10.3389/fphar.2026.1972561

**Published:** 2026-10-26

**Authors:** Swapnil Chavan

**Affiliations:** Bioanalysis and Toxicology Unit, Department of Chemical Process and Pharmaceutical Development, Division Life Sciences, RISE Research Institutes of Sweden, Södertälje, Sweden

**Keywords:** graph edit network, graph neural networks, metabolite prediction, Phase I metabolism, sites of metabolism

## Abstract

**Introduction:**

Predicting the metabolic fate of drug candidates remains a central challenge in pharmaceutical research, with direct implications for efficacy, safety, and pharmacokinetic optimization.

**Methods:**

We present Metabolite‐GEN, an integrated graph neural network framework that predicts SOM and cytochrome P450–mediated reaction class from molecular graph representations, then uses these predictions to enumerate and rerank feasible metabolite structures. The framework was evaluated by five-fold scaffold-stratified cross‐validation on 578 parent–metabolite pairs spanning 12 CYP-mediated reaction classes from the MetXBioDB database, followed by a single evaluation on a scaffold‐disjoint held-out test set of 144 molecules.

**Results:**

In cross‐validation the model achieved mean SOM atom‐ranking top‐1/top‐3/top-5 accuracies of 56.9% ± 6.7%, 78.6% ± 3.2% and 87.5% ± 1.8% (MRR 0.698), reaction class accuracy 37.2% ± 11.4% (weighted F1 0.365), and metabolite structure top‐1/top‐3/top‐5 accuracies of 56.6% ± 4.1%, 73.4% ± 2.4% and 79.1% ± 3.7% (MRR 0.653). On the test set, SOM accuracies were 54.9%, 79.2% and 86.8% (MRR 0.688), reaction class accuracy 31.9% (weighted F1 0.323, macro F1 0.271), and metabolite structure accuracies 53.5%, 72.2% and 75.0% (MRR 0.626).

**Discussion:**

The framework returns explicit metabolite structures rather than atom‐level scores, and the candidate pool for a typical compound is short enough to inspect directly, so the output can be acted on without further filtering—prioritizing compounds for metabolic‐stability screening, flagging reactive metabolite liabilities, and focusing experimental metabolite identification.

## 1 Introduction

The metabolic transformation of xenobiotics is central to drug pharmacokinetics, governing the balance between therapeutic activity and toxicity (Hobbs and Mcllhenny, 1976). Cytochrome P450 (CYP) enzymes are responsible for the oxidative metabolism of large percentage of clinically approved drugs, catalysing reactions including hydroxylation, dealkylation, epoxidation, and oxidation (Brodie et al., 1958; Song et al., 2021). Accurately predicting the metabolic fate of drug candidates early in discovery can flag toxic metabolites, guide structural optimisation, and substantially reduce attrition during preclinical and clinical development (Bugrim et al., 2004; Gajula et al., 2021).

Traditional *in vitro* and *in vivo* metabolite identification approaches are resource-intensive and time-consuming (Castro-Perez, 2007; Li et al., 2024). Computational prediction of metabolic soft spots and biotransformation products offers a rapid, cost-effective complement to experimental work. The evolution of *in silico* tools for predicting cytochrome P450 (CYP)–mediated metabolism began with rule-based expert systems that encoded biochemical knowledge into transformation rules. One of the earliest systems, MetabolExpert (1987) (Darvas, 1987), introduced the concept of computationally enumerating metabolites using curated biotransformation logic. This was followed by hybrid statistical–rule-based approaches such as MultiCASE/META (1994) (Klopman, 1992), which combined structural alerts with probabilistic reasoning. By the late 1990s, more mature and widely adopted expert systems emerged, including METEOR and DEREK (1999) (Greene et al., 1999), providing structured knowledge bases for metabolism and toxicological endpoints. The mid-2000s marked a shift toward structure-based and reactivity-driven prediction with MetaSite (2005) (Cruciani et al., 2005), which integrated molecular interaction fields and accessibility to predict atom-level sites of metabolism. This was complemented by rule-based metabolite generation tools such as SyGMa (2008) (Ridder and Wagener, 2008), and data-driven approaches like MetaPrint2D (2010) (Carlsson et al., 2010) and SMARTCyp (2010) (Rydberg et al., 2010), which estimated metabolic hotspots using statistical occurrence patterns and quantum-chemistry–inspired descriptors.

The following decade saw the integration of machine learning and large-scale data into metabolism prediction. Tools such as IMPACTS (2012) (Campagna-Slater et al., 2012) and FAME (2013) (Kirchmair et al., 2013) introduced faster, ligand-based sites of metabolism (SOM) prediction, while deep learning–based platforms like XenoSite demonstrated improved predictive accuracy across CYP isoforms. Subsequent systems, including Way2Drug (2016) (Rudik et al., 2016), expanded accessibility via web-based prediction of reactive atoms. The field then progressed toward integrated metabolite generation frameworks such as BioTransformer (2019) (Djoumbou-Feunang et al., 2019) and GLORY (2019) (de Bruyn Kops et al., 2019), which combined rule-based and machine learning strategies. More recent developments include CyProduct (2021) (Tian et al., 2021), followed by emerging AI-driven systems such as MetaPredictor (2024) (Zhu et al., 2024), MetaSense (2025) (Meng and Pei, 2025), DeepMetab (2025) (Zhou et al., 2025), and MetaReact (2025) (Wang et al., 2026) – that have progressively integrated rule-based and learned strategies to approach end-to-end metabolite prediction.

Despite this progress, a critical limitation persists across the field: existing tools treat SOM identification, reaction-class assignment, and metabolite structure generation as separate, sequential subtasks. Errors therefore propagate across stages, and the combinatorial space between a predicted SOM and the reaction it undergoes is never resolved as a unit. A site predicted by one module and a reaction type predicted by another can be chemically incompatible, yet nothing in a sequential pipeline prevents the pair from being carried forward into a proposed structure.

Graph neural networks (GNNs) are uniquely suited to close this gap. By operating directly on molecular graph representations—without hand-crafted fingerprinting—architectures such as graph attention networks (GAT) and message-passing neural networks (MPNN) have achieved state-of-the-art performance across computational chemistry. Their atom-level resolution makes them particularly natural for predicting atom-specific properties (SOM), while the same representations can be pooled to predict molecule-level outputs (reaction class).

Here we present Metabolite-GEN (Metabolite Graph-Edit Networks), a framework that brings all three metabolic prediction tasks under a single evaluation and selection protocol. Graph-Edit Networks (GEN) encodes molecular graphs through a multi-head graph attention encoder to produce rich atom-level embeddings, which are decoded by dedicated SOM and reaction-classification heads. Top-k predicted SOMs and reaction classes were combinatorially paired to enumerate candidate metabolite SMILES, which a learned reranking module then scores. Rather than optimizing each stage against its own metric, the complete pipeline is executed on validation data after every training epoch, so the three components are selected together on the quality of the structures they jointly produce. This architecture offers two advantages: every proposed edit is constrained to a SOM–reaction pairing evaluated as a unit rather than assembled from independent predictions, and metabolites are retrieved as explicit ranked structures without *post hoc* filtering. We evaluated Metabolite-GEN on the MetXBioDB database using 5-fold cross-validation and a held-out test set, demonstrating competitive accuracy across all three tasks and establishing Metabolite-GEN as a strong foundation for integrated metabolism prediction in drug discovery.

## 2 Methodology

### 2.1 Dataset

We used the MetXBioDB database as the primary data source, a curated collection of experimentally verified parent-metabolite pairs from the published literature (Wishart et al., 2022). The dataset was processed to retain 722 unique parent molecules with unambiguous metabolite annotations, spanning 12 distinct CYP-mediated reaction classes. Each entry consists of a parent SMILES string, the ground-truth metabolite SMILES, the site of metabolism, and the reaction class label.

The 722 compounds were partitioned into a pre-training pool (578 molecules, 80%) and a held-out test set (144 molecules, 20%) using a scaffold-based split. Where a parent molecule is annotated with more than one experimentally observed metabolite, all of its metabolites are kept together in the same split, so that no metabolite of a test parent can appear in the pre-training set. The pre-training pool was subsequently used in 5-fold cross-validation, with each fold containing approximately 462 training and 116 validation parent-metabolite pairs. The held-out test set was evaluated exactly once, after the final model had been constructed.

Reaction classes represented by fewer than 80 examples were designated as minority classes and addressed by combining a multi-class focal loss with minority-class oversampling, applied to the training split only. Additional training instances were generated by re-encoding each minority-class molecule as randomized SMILES. Because the atom and bond features described in Section 2.2 are computed from the molecular graph rather than from the SMILES string, randomized representations of the same molecule yield an identical chemical graph. The procedure consequently duplicates minority-class graphs rather than introducing new structural variation, and we describe it as oversampling rather than as data augmentation throughout.

Randomized SMILES do, however, permute atom ordering, which would misalign the atom-level SOM labels if the graph were simply reparsed. Persistent RDKit atom-map identifiers are therefore assigned to the source molecule before each randomized representation is generated, SOM labels are transferred by atom identity after reparsing, and each randomized representation is verified to canonicalize back to the original SMILES before its graph is constructed.

### 2.2 Molecular graph representation

Each molecule was represented as a graph G = (V, E), where vertices V corresponded to heavy atoms and edges E corresponded to covalent bonds. Atom features comprised a 19-dimensional vector per atom, formed from 10 generic descriptors and nine engineered metabolic soft-spot descriptors.

Generic descriptors (10): Gasteiger partial charge, mean neighbour Gasteiger charge, valence electron count, TPSA contribution, lone-pair estimate, second-shell heavy-neighbour count, ring count, LogP contribution, molar-refractivity contribution and aromatic-neighbour fraction.

Engineered soft-spot descriptors (9): benzylic carbon flag, allylic carbon flag, tertiary amine nitrogen flag, heteroatom soft-spot flag, linker atom flag, thioether sulphur flag (S-oxidation), alpha-to-nitrogen carbon flag (N-dealkylation), benzylic nitrogen flag (N-oxidation) and sp2 non-aromatic ring-carbon flag (epoxidation).

This partition is used directly in the ablation of Section 2.8, where the nine engineered descriptors are removed and the model is retrained on the 10 generic descriptors alone. Bond features were encoded as a 4-dimensional one-hot vector (single, double, triple, aromatic) and supplied as edge attributes to each graph-attention convolution.

### 2.3 Model architecture

Metabolite-GEN comprises two prediction branches, a deterministic candidate generator, and a learned reranker.

The SOM branch uses a graph attention network (GATv2) encoder with multiple message-passing layers, each with several attention heads, residual connections between layers, and edge-conditioned attention using the bond features of Section 2.2. At each layer, atom features are updated by aggregating information from neighboring atoms using learned attention weights and a nonlinear activation. A linear projection maps each resulting atom embedding to a scalar SOM logit, trained with binary focal loss on atom-level binary SOM labels.

The reaction branch uses a structurally identical GATv2 encoder. Global mean pooling aggregates its atom embeddings into a molecule-level representation, which passes through a two-layer MLP (ELU, dropout) to predict one of 12 reaction classes under multi-class focal loss.

The two branches instantiate separate encoders with separate losses and separate optimizers. They share the atom and bond featurization of Section 2.2 and are coupled through the downstream generation stage and the joint selection criterion of Section 2.5, but no gradient passes between them (see Figure 1).

**FIGURE 1:**
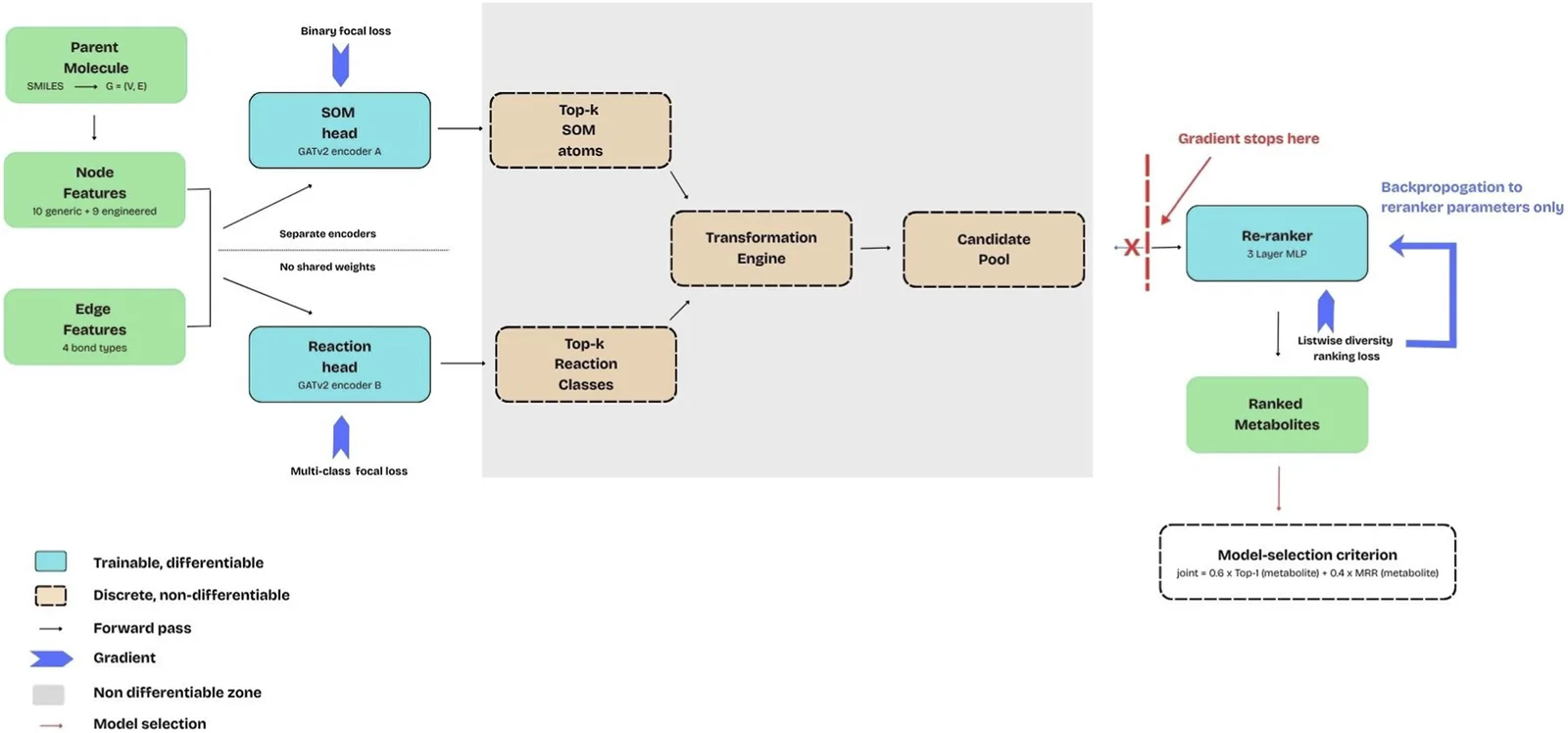
Metabolite-GEN architecture integrating independent prediction branches, deterministic candidate generation, and learned reranking.

Dropout is applied during training, and edges are randomly removed with a fixed probability, to limit overfitting and improve generalization. The depth, width, number of attention heads, dropout rate and edge-drop probability were all determined by the search described in Section 2.6; the selected values are reported with the results.

### 2.4 Candidate generation and metabolite reranker

Given the top-k SOM predictions and top-k reaction class predictions, candidate metabolite SMILES were generated by applying each predicted reaction transformation at each predicted site using a deterministic SMILES transformation engine that encodes the chemistry of the 12 reaction classes. Products that fail RDKit sanitization were discarded, surviving products were canonicalized, and duplicates were collapsed.

Each candidate was scored by a three-layer MLP reranker whose input concatenates the SOM sigmoid probability at the applied site, the reaction softmax probability of the applied class, the ECFP4 fingerprint of the candidate, the mean inter-candidate Tanimoto diversity of the pool, and the normalized SOM and reaction ranks. The reranker was trained with a listwise ranking loss augmented by a diversity-aware term that penalizes pools in which highly ranked candidates were mutually redundant. It was optimized independently of the encoders: top-k selection and rule application were discrete operations, so the ranking loss updates the reranker parameters only.

### 2.5 Training protocol

Metabolite- GEN was implemented in PyTorch 2.9.1 with PyTorch Geometric (v2.7.0) for graph operations. All experiments used an NVIDIA CUDA-enabled GPU with automatic mixed precision (AMP). The AdamW optimiser was used with weight decay 1 × 10^−4^. Learning rates follow cosine annealing from an initial value of 5 × 10^−4^ (SOM/Rxn heads) and 1 × 10^−4^ (reranker) to a minimum of 1 × 10^−5^. Gradient norms are clipped to 5.0 for all components. Training proceeds for up to 300 epochs per fold.

A key methodological element was the use of a pipeline-level validation metric for early stopping. After each training epoch, the complete pipeline is executed on the validation set: the SOM model, reaction model, and reranker were all run in inference mode, candidates were generated and ranked, and each predicted metabolite was compared against all ground-truth successors by (1) exact canonical SMILES match or (2) ECFP4 Tanimoto similarity ≥0.95. The joint score is defined as:

$$joint=0.6*val_{top1}+0.4*val\_MRR$$

where val_top1 is the fraction of validation molecules for which the rank-1 predicted metabolite is a hit, and val_MRR is the mean reciprocal rank of the first hit across all validation molecules. Molecules with empty candidate pools contribute rank ∞ (MRR = 0, top-k miss) to prevent metric inflation by skipping chemically intractable cases. Early stopping halts training after 30 epochs without improvement in the joint score.

Training and evaluation proceed in two stages. Five-fold scaffold-split cross-validation on the pre-training set serves two purposes only: quantifying fold-to-fold stability and fixing the epoch budget for the final model. The final model was then retrained on the complete pre-training set for the mean of the five per-fold best epochs, with no validation split and no early stopping, and applied once to the held-out test set. No fold was promoted to the reported result, and no test-set quantity enters any selection decision. For transparency, each fold model and a five-model probability-averaging ensemble were additionally evaluated on the test set; these were reported as diagnostics and were never used for selection.

### 2.6 Hyperparameter optimization

Architectural hyperparameters were selected by Gaussian-process Bayesian optimization using expected improvement as the acquisition function (GPyOpt). Seven ordinal discrete variables were searched: the number of GATv2 layers (2–5), the number of attention heads (2–8), the hidden width of the SOM branch, the reaction branch and the reranker (each 64, 128 or 256), the dropout rate (0.1, 0.2 or 0.3) and the edge-drop probability (0.1, 0.2 or 0.3). The resulting grid contains 6,804 configurations, which is infeasible to evaluate exhaustively at the cost of a training run per point; Gaussian-process optimization was used because it typically locates a good region of such a space within a few trials. The budget was 50 trials: 10 initial design points followed by 40 Gaussian-process-guided iterations.

Each trial was scored by three-fold scaffold-split cross-validation on the pre-training set, with a budget of 60 epochs and an early-stopping patience of 12 per trial fold. The trial objective was the mean across folds of the per-fold best joint score defined in Section 2.5, so that the search optimized the same pipeline-level criterion used for early stopping rather than a proxy such as SOM accuracy.

The held-out test set was never evaluated during the search. The search was conducted once, on the reference configuration, and the selected values were then held fixed across every ablation variant so that comparisons between variants differ only in the component under test. The selected values are reported in Section 3.1.

### 2.7 Evaluation metrics

SOM prediction performance is reported as top-1, top-3, and top-5 atom-ranking accuracy and mean reciprocal rank (MRR). For each molecule, the true SOM atom indices are ranked against the sigmoid probability scores output by the SOM head; a hit at rank k requires any true SOM atom to appear in the k highest-ranked positions:

$$MRR=\left(\frac{1}{\left|Q\right|}\right)\sum_{i=1}^{\left|Q\right|}\frac{1}{{rank}_{i}}$$

where:

-
Q is the set of queries,
-
${\text{rank}}_{i}$ is the rank position of the first correct answer for the i -th query.

Reaction class prediction is assessed by the weighted F1 score, which accounts for class imbalance. Let C be the set of classes. For each class c ∊ C, F1-score, and Weighted F1-score are defined as:

$${F1\;score}_{c}=\frac{2*{Precision}_{c}*{Recall}_{c}}{{Precision}_{c}+{Recall}_{c}}$$

$${Weighted\;F1\;score}_{c}=\sum_{c∊C}\frac{N_{c}}{N}*{F1\;score}_{c}$$

where:

- TPc, FPc, and FNc are the true positives, false positives, and false negatives for class c ,
- Precisionc = TPc/(TPc + FPc),
- Recallc = TPc/(TPc + FNc),
- Nc the number of compounds in class c ,
- N is total number of compounds

End-to-end metabolite structure accuracy is reported as top-k accuracy (top-1, top-3, top-5), where a prediction is considered correct if the Tanimoto similarity between the predicted and ground-truth metabolite structure equals 1.0 or ≥0.95.

For a set of compounds Q, top-k accuracy is defined as:

$$Top\;k=\left(\frac{1}{\left|Q\right|}\right)\sum_{i=1}^{\left|Q\right|}I\left(y_{i}∊{Ŷ}_{i}^{k}\right)$$

where:

- y_i_ the ground-truth metabolite for the *i*th compound,
- Ŷ^(k)^_i_ is the set of the top k predicted metabolites,
- 𝕀(.) is the indicator function, equal to 1 if the condition is true and 0 otherwise.

### 2.8 Ablation strategy

To identify which components carried the performance of the framework, six variants were trained, each differing from the reference configuration in exactly one component and each following the protocol of Section 2.5 with the optimized hyperparameters of Section 2.6 held fixed: (1) the diversity-aware ranking term was removed, leaving a plain listwise reranker; (2) the nine engineered metabolic soft-spot descriptors were removed, reducing the atom feature vector from 19 to 10 dimensions; (3) the GATv2 encoder was replaced by a two-layer GCN without attention or edge conditioning, with the rule engine and reranker unchanged; (4) a shared-trunk variant was trained in which a single GATv2 encoder fed two lightweight task heads and one optimizer updated it from a weighted sum of both focal losses, so that both task gradients accumulated in the same encoder parameters; (5) the SOM branch was trained alone, with no reaction head, candidate generation, or reranker, and selected on validation SOM MRR; and (6) the reaction branch was trained alone, selected on validation weighted F1.

Variants (5) and (6) isolated the effect of selecting both branches on a single pipeline-level criterion, while variant (4) tested whether the two tasks benefited from a shared representation. Each variant was evaluated on the held-out test set both as the refitted single model and as the five cross-validation fold models, the latter providing the variability estimate against which differences between variants were judged.

## 3 Results and discussion

### 3.1 Optimised hyperparameters

The Bayesian search described in Section 2.6 completed all 50 trials without failure. The selected configuration is given in Table 1.

**TABLE 1:** Hyperparameters of the reference Metabolite-GEN configuration, with searched values selected by Bayesian optimization.

Hyperparameter	Search range	Selected value
GATv2 layers	2, 3, 4, 5	2
Attention heads	2–8	5
Hidden width, SOM branch	64, 128, 256	64
Hidden width, reaction branch	64, 128, 256	64
Hidden width, reranker	64, 128, 256	256
Dropout rate	0.1, 0.2, 0.3	0.1
Edge-drop probability	0.1, 0.2, 0.3	0.1

The purpose of the search was to fix a single architectural setting in advance of any test-set evaluation. These values were then held identical across the reference model and all six ablation variants, so that the component-wise comparisons of Section 3.4 differ only in the component under test and no variant has been individually tuned.

### 3.2 Cross-validation performance of reference model

Metabolite-GEN was evaluated using five-fold scaffold-split cross-validation. As summarized in Table 2 and Supplementary Table S2, SOM MRR ranged from 0.647 to 0.741 (mean 0.698 ± 0.039), reflecting how highly the correct SOM atom was ranked overall; higher MRR values indicate that the correct atom was more frequently positioned near the top of the prediction list. Top-1 SOM accuracy ranged from 46.55% to 64.35% across validation folds (mean 56.9% ± 6.7%), indicating that the model identified the SOM atom as its top-ranked prediction for 54–74 validation molecules per fold. For example, in Fold one the correct SOM atom was ranked first for 54 of 116 validation molecules. Top-3 accuracy ranged from 73.28% to 81.90% (85–95 molecules) and top-5 from 85.22% to 89.66% (98–104 molecules). The correct SOM atom was therefore generally among the top five predictions even when it was not ranked first.

**TABLE 2:** Five-fold cross-validation results for SOM, reaction and metabolite predictions.

Task	Metric	Mean ± SD	Range
SOM	Top-1 accuracy (%)	56.9 ± 6.7	46.6–64.3
SOM	Top-3 accuracy (%)	78.6 ± 3.2	73.3–81.9
SOM	Top-5 accuracy (%)	87.5 ± 1.8	85.2–89.7
SOM	MRR	0.698 ± 0.039	0.647–0.741
Reaction	Accuracy (%)	37.2 ± 11.4	18.3–48.7
Reaction	Weighted F1	0.365 ± 0.108	0.177–0.456
Metabolite	Top-1 accuracy (%)	56.6 ± 4.1	50.4–60.3
Metabolite	Top-3 accuracy (%)	73.4 ± 2.4	70.7–76.5
Metabolite	Top-5 accuracy (%)	79.1 ± 3.7	75.9–83.5
Metabolite	MRR	0.653 ± 0.032	0.611–0.692

Reaction-class prediction achieved top-1 accuracies of 18.26%–48.70% across the five folds (mean 37.2% ± 11.4%), corresponding to 21–56 of the 115–116 validation molecules in each fold. Weighted F1 ranged from 0.177 to 0.456, with a mean of 0.365 (Table 2). The weighted F1 score provides a complementary measure of classification performance by accounting for both the correctness of the predicted reaction classes and the distribution of molecules across classes, with higher values indicating better overall performance. In contrast to the SOM and metabolite tasks, performance on this task varied considerably between folds. Scaffold-based partitioning balances chemical series rather than biotransformation types, so a fold may contain validation molecules drawn from reaction classes that are sparsely represented in its own training partition. Reaction-class performance is consequently reported as mean ± SD throughout, and figures from any single partition should be interpreted with caution.

Averaged across the five folds, the correct metabolite appeared among the top five ranked candidates for 79.1% ± 3.7% of validation molecules, corresponding to 88–96 of the 115–116 molecules in each fold. The mean reciprocal rank of the first correct candidate was 0.653 ± 0.032 (range 0.611–0.692), indicating that when the correct structure was retrieved it was typically placed at or near the top of the list rather than deep in the pool. Exact canonical SMILES matches at rank one accounted for 46.4% ± 5.7% of validation molecules, corresponding to 42–58 molecules per fold, while a further 10.2% ± 2.3% (9–16 molecules per fold) were recovered only by structural similarity. Roughly four in five rank-1 recoveries were therefore exact rather than approximate.

### 3.3 Test set performance of reference model

The reference model was refitted on all 578 pre-training set molecules and applied once to the 144 held-out test molecules. Because a single refitted model gives no indication of how much its performance depends on the particular training partition, the five cross-validation models were also applied individually to the same test set, and a five-model ensemble was formed by averaging SOM and reaction probabilities before candidate generation. All three views are reported in Table 3; the refitted model is the reported result and the other two are diagnostics. Supplementary Tables S9–S11 give the corresponding refitted, fold-model and ensemble results for every variant.

**TABLE 3:** Held-out test set (n = 144) performance of the reference model: the refitted model, the five cross-validation models applied individually, and their ensemble.

Metric	Refitted model	Fold models (mean ± SD)	Ensemble of 5 folds
SOM top-1 (%)	54.9	56.7 ± 2.6	61.8
SOM top-3 (%)	79.2	77.4 ± 1.6	77.8
SOM top-5 (%)	86.8	85.1 ± 2.7	87.5
SOM MRR	0.688	0.693 ± 0.017	0.728
Metabolite top-1 (%)	53.5	49.0 ± 4.2	56.9
Metabolite top-3 (%)	72.2	70.1 ± 4.4	75.0
Metabolite top-5 (%)	75.0	74.3 ± 5.0	77.8
Metabolite MRR	0.626	0.595 ± 0.045	0.658

On SOM ranking, the refitted model placed an annotated site at rank 1 for 79 of 144 test molecules (54.9%), within the top three for 114 (79.2%) and within the top five for 125 (86.8%), with MRR 0.688. The five fold models gave 56.7% ± 2.6%, 77.4% ± 1.6% and 85.1% ± 2.7% (MRR 0.693 ± 0.017) on the same molecules, so the refitted model falls within one standard deviation of the fold mean on every SOM metric.

The metabolite prediction followed the same pattern. The refitted model recovered an annotated metabolite at rank 1 for 77 of 144 molecules (53.5%), within the top three for 104 (72.2%) and within the top five for 108 (75.0%), with MRR 0.626. The fold models averaged 49.0% ± 4.2%, 70.1% ± 4.4% and 74.3% ± 5.0% (MRR 0.595 ± 0.045). The ensemble outperformed both other views on all metabolite metrics: 82 molecules at rank 1 (56.9%), 108 within the top three (75.0%), 112 within the top five (77.8%) and MRR 0.658. On SOM ranking it improved top-1 (61.8%), top-5 (87.5%) and MRR (0.728).

### 3.4 Component-wise ablation

As per Section 2.8, six variants were trained under the protocol of Section 2.5, each differing from the reference in a single component (Refer Table 4). Full cross-validation results for the reference model and each variant are given in Supplementary Tables S2–S8.

**TABLE 4:** Component-wise ablation on the held-out test set (n = 144). SOM and metabolite values are mean ± SD across the five cross-validation models applied individually to the test set; reaction weighted F1 is from the refitted model.

Variant	SOM top-1 (%)	SOM top-5 (%)	Reaction weighted F1	Metabolite top-1 (%)	Metabolite top-5 (%)
Reference model	56.7 ± 2.6	85.1 ± 2.7	0.323	49.0 ± 4.2	74.3 ± 5.0
No diversity ranking	57.1 ± 1.3	84.6 ± 2.6	0.323	49.6 ± 3.9	74.7 ± 5.2
No engineered descriptors	51.8 ± 4.1	87.1 ± 2.5	0.246	47.2 ± 2.8	73.5 ± 3.2
No GATv2 (Simple GCN)	50.1 ± 2.9	80.7 ± 2.3	0.167	41.9 ± 4.3	65.6 ± 5.8
SOM branch alone	56.4 ± 1.3	85.0 ± 0.8	n/a	n/a	n/a
Reaction branch alone	n/a	n/a	0.315	n/a	n/a
Shared GATv2 trunk	26.5 ± 3.1	76.1 ± 3.0	0.279	47.1 ± 2.6	67.1 ± 2.3

The encoder contributes most. Replacing GATv2 with a simple GCN of two layers, while keeping the rule engine and the reranker unchanged, had reduced every metric at once. What the framework depends on is the attention-based encoder, not the way the two tasks are combined and not the ranking objective.

The diversity-aware ranking term has no measurable effect. Its removal leaves site prediction and reaction classification unaffected, as expected since neither branch sees the ranking loss, and the metabolite differences are smaller. There is no evidence that the diversity-aware ranking term improves accuracy. The reason to keep it would be the property it was designed to produce, a top N list with less redundancy, rather than better ranking.

The engineered descriptors sharpen atom level ranking. Removing them lowers SOM top 1 accuracy while leaving top 5 slightly higher but within one standard deviation, so the generic descriptors still find the reactive region of the molecule but rank atoms within it less precisely. Their effect on downstream metabolite accuracy is not resolved at this sample size.

Coupling the two branches through the selection criterion alone changes neither task. Two variants show this. When the SOM branch is trained on its own, it performs the same as it does inside the reference framework, at both top-1 and top-5. When the reaction branch is trained on its own, it reaches the same weighted F1 as the reference framework.

A shared encoder, by contrast, degrades site prediction. When a single encoder holds most of the model’s parameters and learns both tasks at once, site prediction suffers badly: top-1 accuracy drops sharply, and even top-5 accuracy falls below that of the reference framework.

### 3.5 Error decomposition

The pipeline can fail at three points: the correct site or reaction is not ranked highly enough/predicted correctly to be used, the transformation engine does not produce the correct structure from the site and reaction pairs it is given, or the correct structure is produced but is not ranked into the top k. Candidate pool recall separates the last of these from the first two, and Table 5 and Supplementary Table S12 reports the decomposition for the refitted reference model on the 144 held out test molecules. For 110 molecules (76.4%) the candidate pool contained a correct metabolite, and no molecule produced an empty pool. Of these, 77 were ranked first (53.5% of the test set) and 108 were ranked within the top five (75.0%). Only two molecules therefore had a correct metabolite in the pool but outside the top five, while 33 had one in the pool but not at rank 1. The remaining 34 molecules (23.6%) never received a correct candidate at all.

**TABLE 5:** Error decomposition for the reference model (refitted) on the held-out test set (n = 144).

Stage	Molecules	% of test set
Correct metabolite present in candidate pool	110	76.4
— and ranked 1st	77	53.5
— and ranked within top 5	108	75.0
— in pool but outside top 5 (recoverable by better ranking)	2	1.4
— in pool but not at rank 1 (recoverable by better ranking)	33	22.9
Correct metabolite never generated (upstream failure)	34	23.6
Empty candidate pool	0	0.0
Upper bound on accuracy from ranking alone	110	76.4

These counts place an upper bound on what better ranking can achieve. Because a reranker can only reorder the enumerated candidates it is given, the best attainable accuracy at any rank is the pool recall of 76.4%. A perfect reranker, one that always placed a pool hit at rank 1, would raise top 1 accuracy from 53.5% to 76.4% but would raise top 5 accuracy by less than two percentage points, since almost every correct structure already present in the pool is ranked within the top five. Improving the ranking stage is therefore worth at most a few points at top 5, whereas improving candidate generation is worth up to 23.6. The ablation in Section 3.4 points the same way: the variant that changed only the ranking objective left pool recall unchanged, while the two variants that reduced pool recall most were also those that reduced metabolite accuracy most. Within the upstream stages, reaction classification is the weaker of the two on this test set, and it is where we would direct further work.

Per-class F1 across the five cross-validation folds of the reference model is reported in Supplementary Table S13, and the corresponding confusion matrix in Supplementary Table S14. Performance falls into three bands. A higher-performing group, led by O-dealkylation, N-dealkylation, S-oxidation, N-hydroxylation and aromatic hydroxylation, is predicted in all or nearly all folds. A moderate group, including alicyclic and aliphatic hydroxylation, dehydrogenation and epoxidation, is recognized in some folds and missed entirely in others. A low-performing group—N-oxidation, terminal/penultimate hydroxylation and C-oxidation—is almost never predicted correctly. The classes the model learns most reliably are those best represented in the training data, and several correspond to chemically distinctive motifs flagged by a dedicated atom descriptor, such as the thioether sulphur or the carbon adjacent to nitrogen described in Section 2.2. The confusion matrix shows which classes absorb the misassignments from the weaker ones. In practice the model identifies the correct reaction type as its single best guess for only about a third of compounds but ranks it within its top five for almost all of them. Because candidates are enumerated from the top-ranked sites and the top-ranked reaction types together, the correct transformation is usually available to the generator even when it was not the first choice.

### 3.6 Advantages and limitations of Metabolite-GEN

Table 6 compares our test set result with published figures for four established tools, using the criterion most often reported in this field: whether at least one experimentally observed metabolite appears among the top five predicted structures. We show all three evaluations of our model. An important caveat applies to this comparison. We did not run any of the four tools ourselves. Their figures come from publications (Ridder and Wagener, 2008; Zhu et al., 2024; Litsa et al., 2020; de Bruyn Kops et al., 2021), where they were measured on different sets of compounds, using different training and test splits, and in some cases a different rule for deciding when a predicted structure counts as correct. Because of this, the table shows roughly where our framework sits among existing methods, but it cannot show that one tool is better than another. With that in mind, our reported model recovers a known metabolite within its top five candidates for 75.0% of test compounds, and the ensemble for 77.8%. This places Metabolite-GEN among the established tools rather than clearly ahead of or behind them: higher than the figures reported for GLORYx and SyGMa, and close to those for MetaTrans and MetaPredictor. Settling the question would require running all the tools on the same set of compounds, which has not yet been done.

**TABLE 6:** Published performance of related tools on the “at least one metabolite correct” criterion at top-5.

Tool	At least one metabolite in top 5 (%)	References
Metabolite-GEN, five model ensemble	77.8	n/a
Metabolite-GEN, refitted model	75.0	n/a
Metabolite-GEN, five fold models	74.3 ± 5.0	n/a
MetaPredictor	77.0	Zhu et al. (2024)
MetaTrans	72.6	Litsa et al. (2020)
SyGMa	68.1	Ridder and Wagener (2008)
GLORYx	60.7	de Bruyn Kops et al. (2021)

#### 3.6.1 Correctly predicted example: clobazam

Clobazam is a 1,5-benzodiazepine anticonvulsant that undergoes predominant CYP-mediated aromatic hydroxylation on its pendant phenyl ring. Metabolite-GEN’s rank-1 prediction correctly identifies atom 11 (a para-carbon of the unsubstituted phenyl substituent) as the primary SOM, matching the annotated aromatic hydroxylation at that position, and the corresponding candidate structure is ranked first in the pool (Figure 2). Atom indices follow canonical SMILES ordering. Figure 3 shows the integrated gradients (IG) map for the same molecule. The highest-attributed atoms lie on the pendant phenyl ring, while the benzodiazepine scaffold and the chlorophenyl ring contribute less. We present this as a description of the model’s internal state rather than as support for the prediction: attribution is computed from the same head that produced the prediction, and attribution agrees with annotated sites only marginally more often than a random ordering of atoms (see Section 3.6.3). The agreement seen here is therefore an individual observation, not a property that holds generally.

**FIGURE 2:**
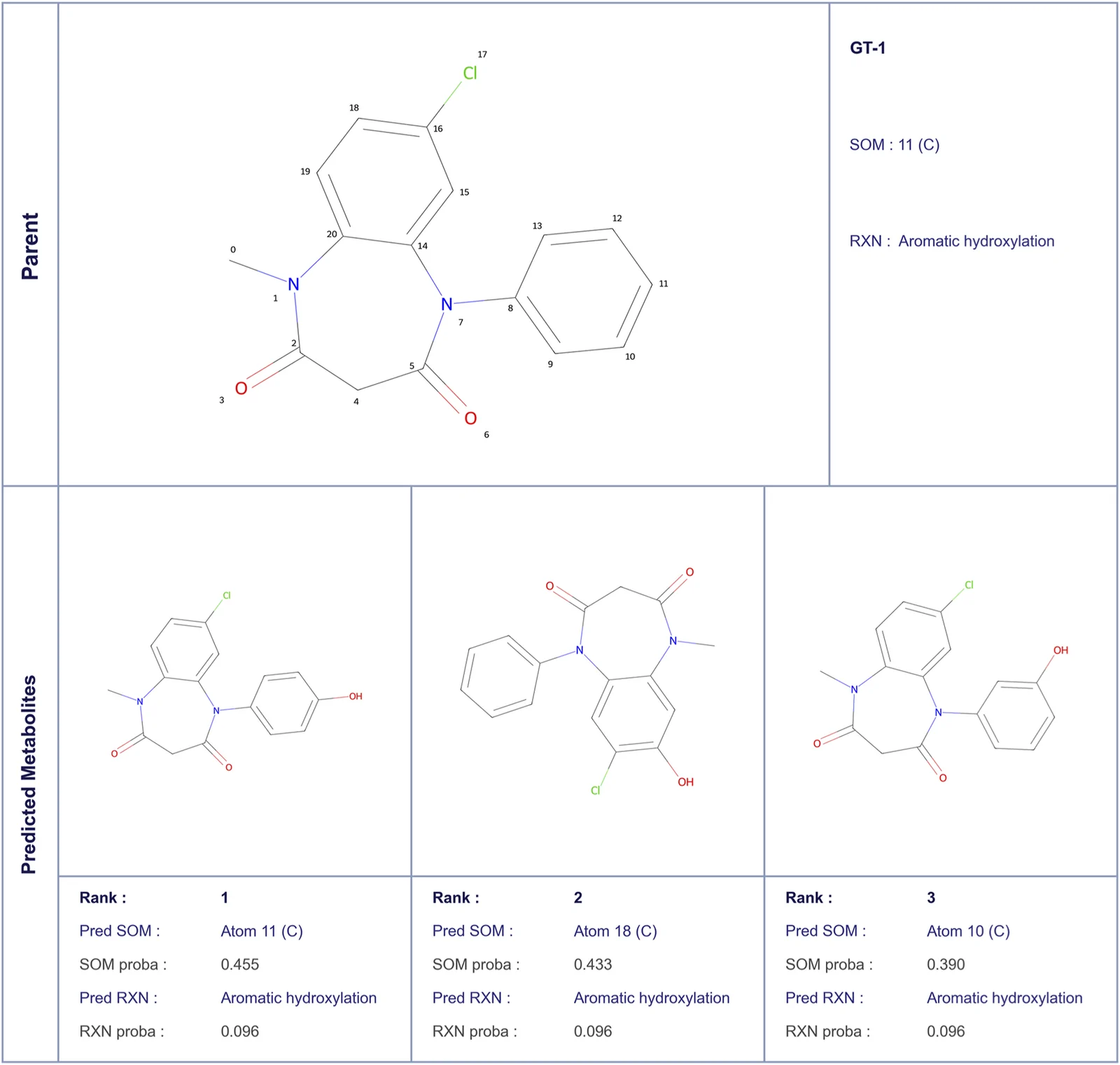
GEN metabolite prediction for clobazam (correctly predicted).

**FIGURE 3:**
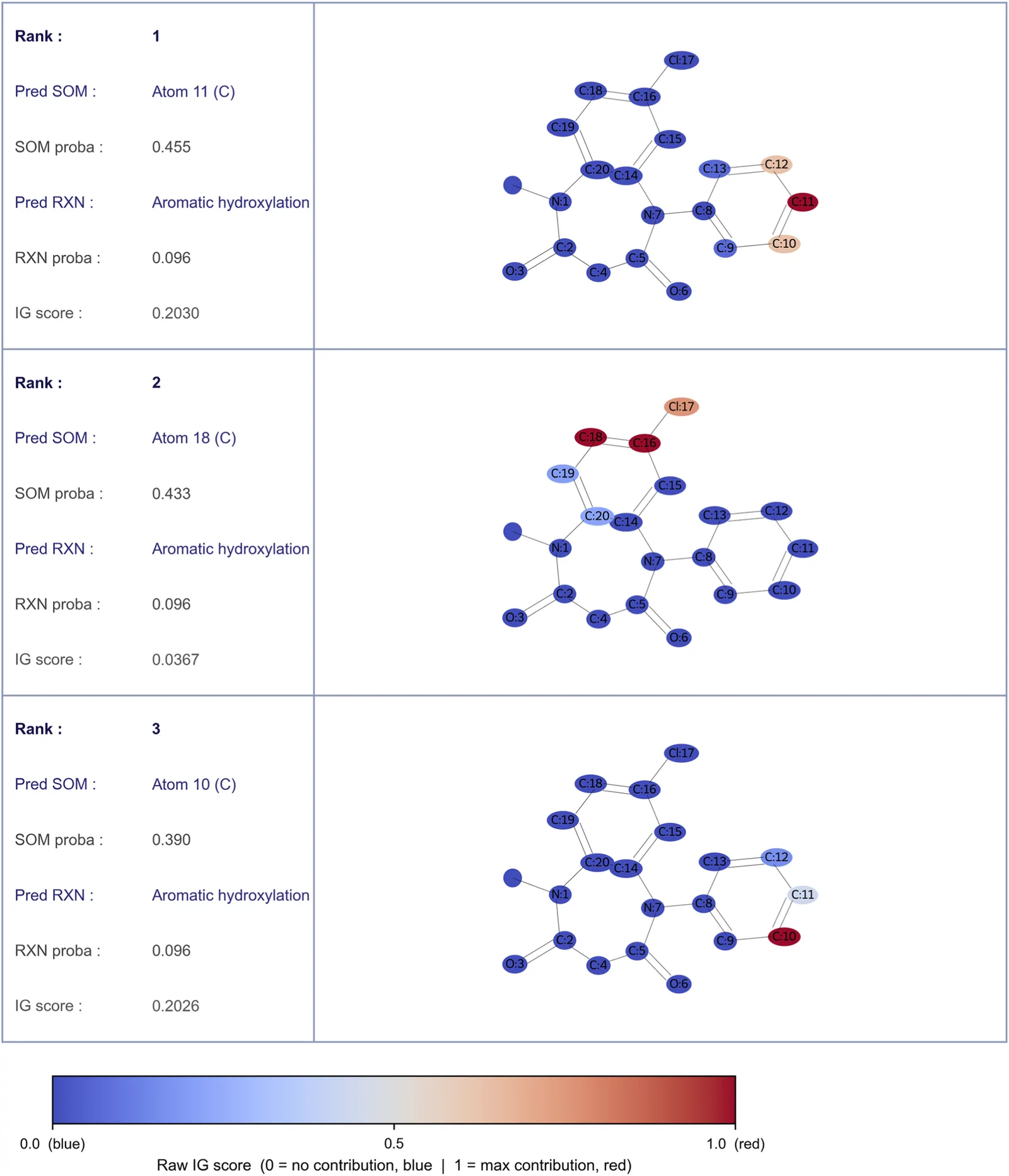
GEN integrated gradients (IG) interpretability for clobazam (correctly predicted).

#### 3.6.2 Incorrectly predicted example: N-nitrosopyrrolidine

N-Nitrosopyrrolidine (NPYR) is a small cyclic nitrosamine whose principal metabolic pathway is CYP-mediated α-hydroxylation at the carbon adjacent to the nitroso nitrogen (atom 6 in the numbering scheme used here), ultimately leading to ring-opening and DNA-alkylating intermediates. Metabolite-GEN’s rank-1 through rank-5 predictions all fail to identify atom 6 as the SOM: the model instead proposes atoms 3 and 4 (ranks 1–4) and atom 2 (rank 5). The correct reaction class, aliphatic hydroxylation, does not appear among the five ranked predictions either; the closest proposal is alicyclic hydroxylation at ranks 1 and 2, a related class applied at the wrong position, followed by C-oxidation at ranks 3 and 4 and N-dealkylation at rank 5 (Figure 4). Because no predicted site–reaction pair corresponds to the true transformation, the correct metabolite never enters the candidate pool, and no reranking could have recovered it—an instance of the upstream failure mode quantified in Section 3.5. Two factors plausibly compound here: the pyrrolidine ring is near-symmetric, so neighbourhood aggregation produces similar embeddings for chemically similar ring carbons, and nitrosamines are sparsely represented in MetXBioDB. Figure 5 shows comparable attribution across several ring carbons, which is consistent with the first of these but does not establish it.

**FIGURE 4:**
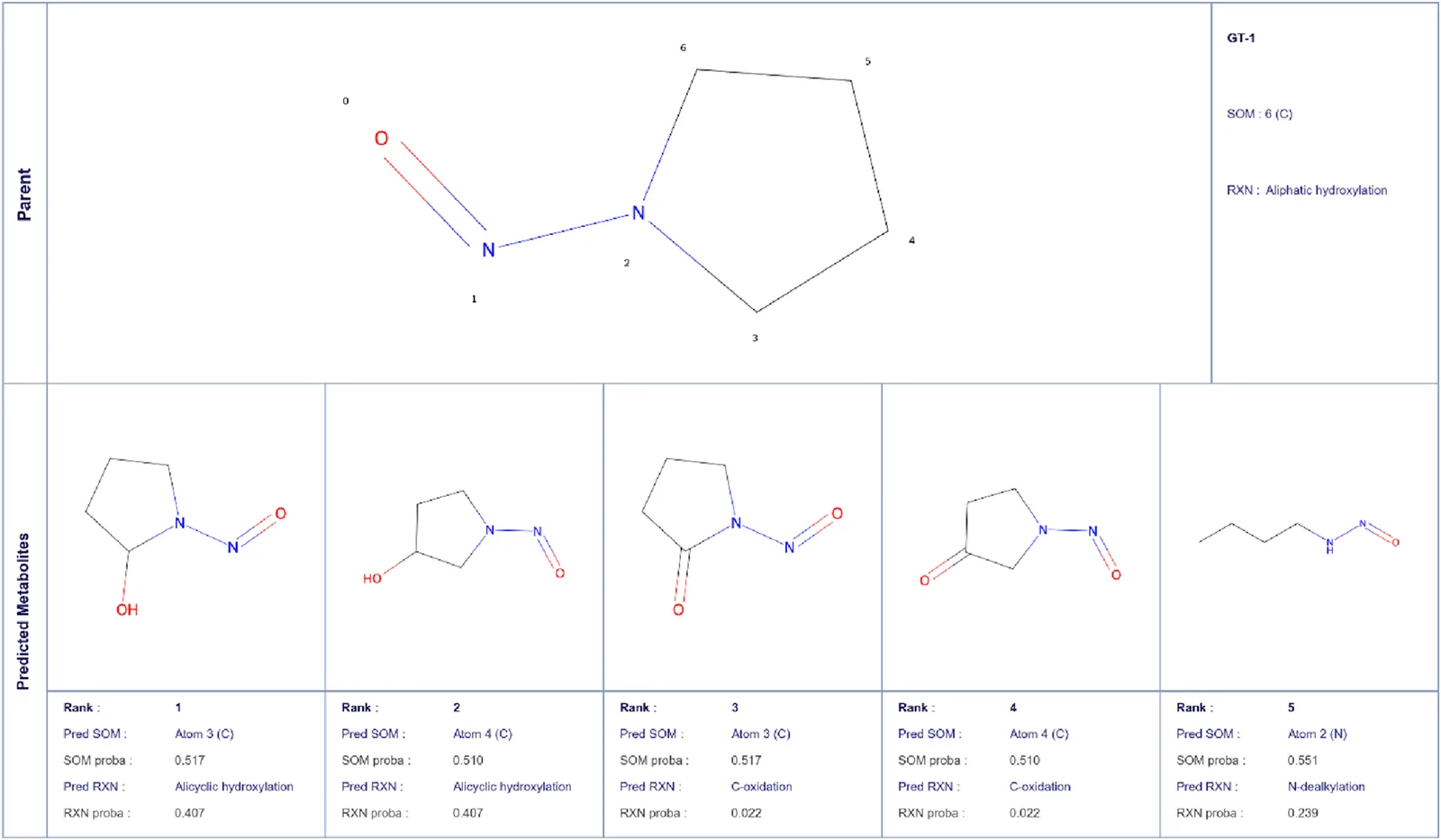
GEN metabolite prediction for N-nitrosopyrrolidine (incorrectly predicted).

**FIGURE 5:**
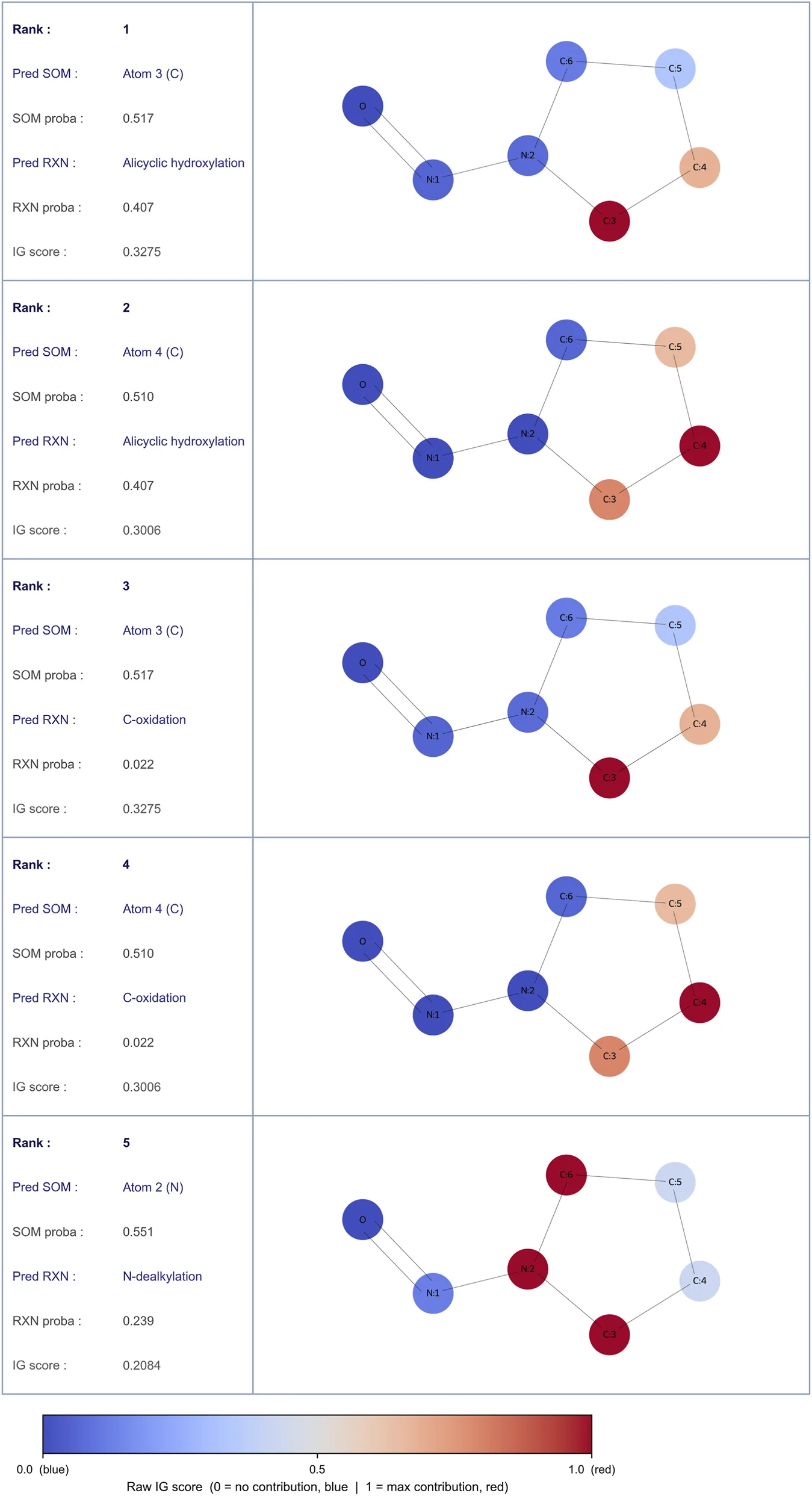
GEN integrated gradients (IG) interpretability for N-nitrosopyrrolidine (incorrectly predicted).

#### 3.6.3 Quantitative analysis of attribution quality

Attribution quality of the refitted reference model was evaluated across all 144 held-out molecules by ranking the atoms of each molecule by IG magnitude and comparing the ranking against the annotated sites of metabolism. The highest-attributed atom was an annotated site for 7.6% of molecules, against 7.5% expected from a random atom ordering; the corresponding top-3 and top-5 values were 29.2% and 47.9% against random baselines of 21.1% and 33.2%, with an atom-level ROC AUC of 0.58. Attribution therefore carries weak information about annotated chemistry and is a substantially poorer guide to it than the model’s own site ranking. We report integrated gradients as a description of model internals and draw no chemical conclusions from it.

#### 3.6.4 Generalization to novel scaffolds

The scaffold-based split was used to reduce structural overlap between the pre-training and held-out test sets. However, all compounds originate from MetXBioDB and therefore share the same overall data source and curation framework. The present results should therefore not be interpreted as demonstrating source-independent external generalization. Instead, they assess performance on structurally distinct compounds within the chemical space represented by MetXBioDB. This limitation is particularly relevant given the relatively small dataset and uneven representation of some reaction classes. Performance may be lower for scaffolds or metabolic transformations that are poorly represented in the current data. Scaffold novelty also does not necessarily imply mechanistic novelty, as structurally different molecules may undergo similar CYP-mediated reactions. Conversely, related scaffolds may still differ in their preferred sites of metabolism. Future work will focus on validating Metabolite-GEN on a suitably harmonized external dataset to assess its robustness beyond the MetXBioDB chemical space.

## 4 Conclusion

We have presented an integrated graph neural network framework for the prediction of sites of metabolism, reaction classes, and metabolite structures. In 5-fold cross-validation on 578 parent–metabolite pairs from MetXBioDB, the model achieved mean top-1, top-3, and top-5 metabolite structure accuracies of 56.6% ± 4.1%, 73.4% ± 2.4%, and 79.1% ± 3.7%, respectively. On a held-out test set of 144 molecules with scaffolds absent from the pre-training set, Metabolite-GEN, the refitted model, achieved top-1, top-3, and top-5 metabolite structure accuracies of 53.5%, 72.2%, and 75.0%, respectively. These results demonstrate the potential of Metabolite-GEN as a baseline for metabolite prediction.

Removing components one at a time showed that the graph attention encoder contributes most, a simpler encoder costing roughly nine percentage points of metabolite top-5 accuracy, while forcing both tasks to share one encoder markedly degraded site prediction and the diversity term in the ranking step had no measurable effect. The error analysis was equally informative: a correct metabolite reached the candidate list for three-quarters of test compounds and was almost always ranked within the top five, so the limitation lies in generating the right structures rather than in ordering them, and broader coverage of biotransformation chemistry is the most promising route to improvement.

Beyond its predictive performance, Metabolite-GEN has potential applications in pharmaceutical research and predictive toxicology, where early assessment of metabolic liability can support compound optimisation and safety evaluation. Reactive metabolites, including electrophilic species, quinones, epoxides, and acyl intermediates, may contribute to adverse drug reactions. However, their identification often requires resource-intensive experimental studies. By generating ranked and structurally explicit metabolite hypotheses directly from molecular graph representations, Metabolite-GEN could help identify potential metabolic and reactive-metabolite liabilities at an early stage of compound evaluation. This information may assist chemists in prioritising compounds and guiding structural optimisation before undertaking more resource-intensive experimental investigations. Thus, Metabolite-GEN provides a potential decision-support tool for integrating metabolic prediction into early drug-discovery and safety-assessment workflows.

## Data Availability

Publicly available datasets were analyzed in this study. This data can be found here: https://zenodo.org/records/13235312.
